# Copy number-based quantification assay for non-invasive detection of PVT1-derived transcripts

**DOI:** 10.1371/journal.pone.0226620

**Published:** 2019-12-26

**Authors:** Gargi Pal, Olorunseun O. Ogunwobi

**Affiliations:** 1 Department of Biological Sciences, Hunter College of The City University of New York, New York, NY, United States of America; 2 Hunter College Center for Cancer Health Disparities Research, New York, NY, United States of America; 3 Joan and Sanford I. Weill Department of Medicine, Weill Cornell Medicine, Cornell University, New York, NY, United States of America; Southern Illinois University School of Medicine, UNITED STATES

## Abstract

**Background:**

One of the most important susceptibility loci for cancer is the 8q24 human chromosomal region. The non-protein coding gene locus plasmacytoma variant translocation 1 (PVT1) is located at 8q24 and is dysregulated in prostate cancer. PVT1 gives rise to multiple transcripts which may have different functions. Here, we describe a real-time quantitative polymerase chain reaction (qPCR)-based assay for copy number-based quantitation of PVT1 exons 4A, 4B, and 9 to enable accurate, reproducible, and quantifiable detection.

**Methods:**

PVT1 exons 4A, 4B, and 9 were cloned into a plasmid vector to create standards for subsequent creation of linear standard curves representing a broad range of concentrations. PCR was carried out using SYBR-Green signal detection to quantify PVT1 exons 4A, 4B, and 9. The efficacy of this assay was evaluated by using it to detect these transcripts in prostate epithelial and prostate cancer cell lines, normal and cancerous human prostate tissues, human serum, mouse plasma, and urine samples.

**Results:**

The results indicate that the assay can be used to quantify both low and high copy numbers of PVT1-derived transcripts. This is the first report of a copy number-based quantification assay for non-invasive detection of PVT1 derived transcripts.

**Conclusions:**

This novel assay holds promise for routine non-invasive testing in diseases where PVT1 is dysregulated.

## Introduction

**Fig 1:**
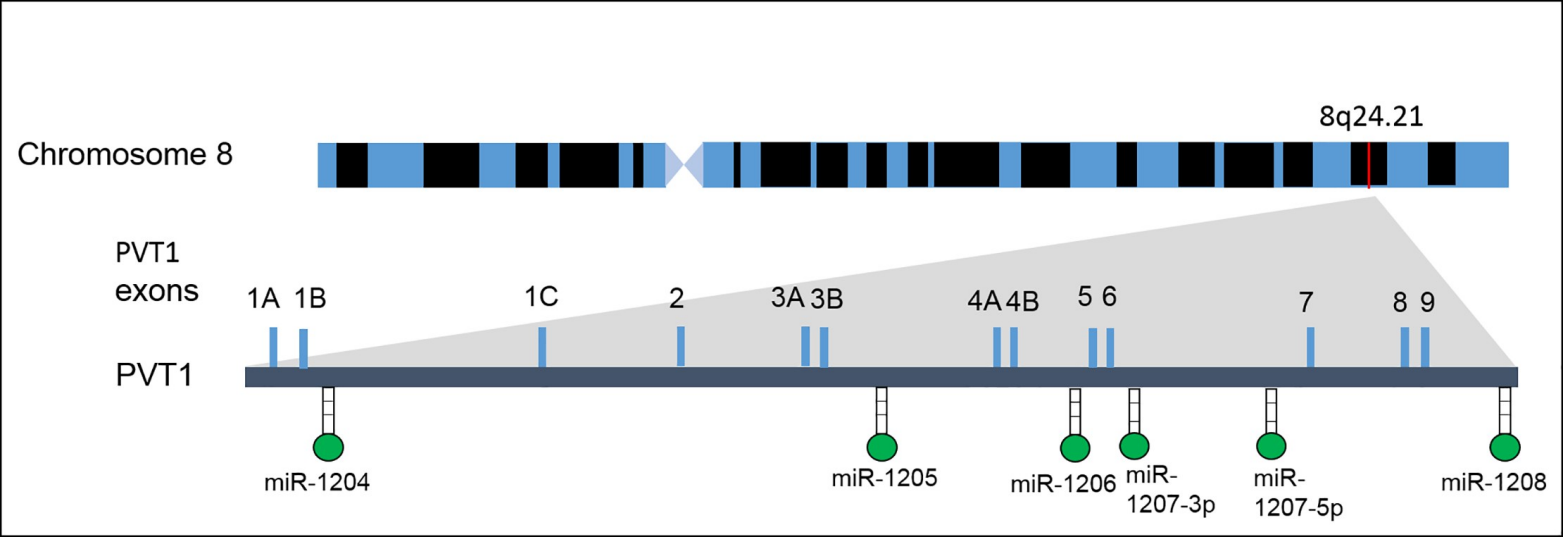
Schematic diagram of PVT1 gene structure). **Its association with factors like cell differentiation, metastatic disease, overall survival, and tumor stage has been widely reported**.

To gain a better understanding of the mechanisms and processes involved with factors involved in the growth and progression of cancers, attention has been turned to long non-protein coding RNAs (lncRNAs) [1]. LncRNAs are defined as endogenous cellular RNAs that have a size of more than 200 nucleotides, and that do not possess an extended open reading frame [2]. Plasmacytoma variant translocation 1 (PVT1), a non-protein coding gene encoded in humans, gives rise to a highly conserved lncRNA. It is located 50 kb downstream of myelocytomatosis (MYC) oncogene at the 8q24.21 chromosomal region. It has a size of over 300 kb and expresses several alternatively spliced non-protein coding transcripts and microRNAs [3, 4] (Fig 1)

Prostate cancer (PCa) is the most commonly diagnosed male cancer in the United States [5], and the most commonly diagnosed male cancer in 103 countries of the world [6]. Globally, in 2015 alone, 1.6 million people were diagnosed with PCa and based on current demographic trends, it is possible that the number of men diagnosed with PCa annually will increase [6]. In PCa, PVT1 has been found to have increased expression in comparison to normal prostate tissue [7,8]. PCa is a complex disease, with several risks factors [9]. PCa is the most common non-cutaneous cancer and the second leading cause of cancer-related death for American males, exceeded only by lung cancer [10]. PCa is also the leading cancer in terms of incidence and mortality in men from Africa and the Caribbean, while native Japanese and Chinese populations have a low risk [11,12]. Screening for and management of early prostate cancer is one of the most challenging and controversial issues in all of medicine [13].

Prostate specific antigen (PSA) has been widely used as a prostate cancer biomarker for many years. However, a major drawback of PSA is its lack of specificity, which results in a high negative biopsy rate [13–15]. Owing to its restricted expression profile, PCA3 RNA serves as a useful biomarker for prostate cancer. The PCA3 score has a higher specificity and a better predictive value than serum PSA, although its sensitivity is lower [15,16]. Consequently, a new assay which accurately diagnoses aggressive PCa, and distinguishes between indolent and aggressive PCa is urgently needed.

The aberration of PVT1 is associated with an increased copy number, upregulation, or over expression of PVT1 in different malignancies including cervical cancer, bladder cancer, colorectal cancer, gastric cancer, hepatocellular carcinoma, and lung cancer [4]. Dysregulation of PVT1 is also associated with breast and ovarian cancer, acute myeloid leukemia and Hodgkin’s lymphoma, vitiligo, and asthma [17].

PVT1 plays an oncogenic role in prostate cancer and knockdown of PVT1 inhibits prostate cancer growth both *in vivo* and *in vitro* and promotes cell apoptosis [18]. Downregulation of lncRNA PVT1 expression inhibits the proliferation, mobility and colony formation abilities of prostate cancer cells [19].

Quantitative reverse transcriptase polymerase chain reaction (qPCR) has become a diagnostic methodology of choice due to its sensitivity and rapid turnaround time. It has been widely used for the detection of several diseases, such as hepatitis B, hepatitis C, enterovirus, and several others, but there is no report of any diagnostic tool that performs quantification of PVT1 transcripts [20–24].

PVT1 is a 1,957 bp linear lncRNA encoded by human PVT1 gene, which expresses several alternatively spliced non protein coding transcripts [25–28]. PVT1 has multiple exons that make separate transcripts which may have different functions [29,30]. We and others are progressively uncovering the fact that these transcripts of PVT1 are differentially expressed in various cancers, and may have different functions [27–33]. Our lab has previously reported that PVT1 exons 4A, 4B, are 9 are significantly overexpressed in aggressive PCa [27–32]. Due to our focused research interest in PCa, we have decided to focus on PVT1 exons 4A, 4B, and 9 because of their potential role as biomarkers for prostate cancer (PCa). In the present study, we focus on developing an assay of transcripts derived from PVT1 exons 4A, 4B, and 9, which have been previously described to be potential biomarkers of prostate cancer [29,31,32]. This assay is likely to have applications in all the diseases characterized by dysregulation of PVT1 [3,17].

In this study, we describe the development and validation of a qPCR-based method to measure the absolute concentrations of PVT1 exons 4A, 4B, and 9 in cell lines, tissues, human serum, mouse plasma, and human urine. This assay is based on the specific amplification of PVT1 exons 4A, 4B, and 9 using primers that amplify PVT1 exons 4A, 4B, and 9, standards that enable quantification and detection in real-time with SYBR Green dye. This assay is able to quantify both low and high copy numbers of PVT1 exons 4A, 4B, and 9. We anticipate future investigations to determine if this novel assay is superior to PSA and PCA3 tests, and if its use of a panel of biomarkers makes it more effective in accurately diagnosing aggressive PCa, and distinguishing between indolent and aggressive PCa.

## Materials and methods

### Human subjects research

The City University of New York Institutional Review Board approved the study (approval number 2017–1144). Informed consent was obtained whenever required by the IRB-approved protocol.

### Animal research

Animal study was approved by Weill Cornell Medicine Institutional Animal Care and Use Committee (IACUC) (approval number 2015–0038). Whenever necessary, euthanasia was performed using carbon dioxide displacement, as approved.

### Creation of assay standards

Double stranded DNA (dsDNA) of PVT1 exons 4A, 4B, and 9 were synthesized, and each of these dsDNAs was amplified by polymerase chain reaction. Three sets of custom-designed primers (Table 1) were used to amplify exon 4A (301bp), exon 4B (130bp), and PVT1 exon 9 (273bp).

**Table 1:** List of primer sequences of PVT1 exons.

Primer name	Primer sequence 5’– 3’
PVT1 exon 4A-F	ATTAAGCTTAGTCTCACTCTGTGGTCCAGG
PVT1 exon 4A-R	ATTGGATCCCTGGACTCTTCAAAAATGTCA
PVT1 exon 4B-F	ATTAAGCTTAATCCTGTTACACCTGGGATT
PVT1 exon 4B-R	ATTGGATCCCTTAATTCTCCAATCTCAAAA
PVT1 exon 9-F	ATTAAGCTTGTTTTTTGCATGTCTGACACC
PVT1 exon 9-R	ATTGGATCC AGTAGAAAAAGAATTTAATAG

**Fig 2:**
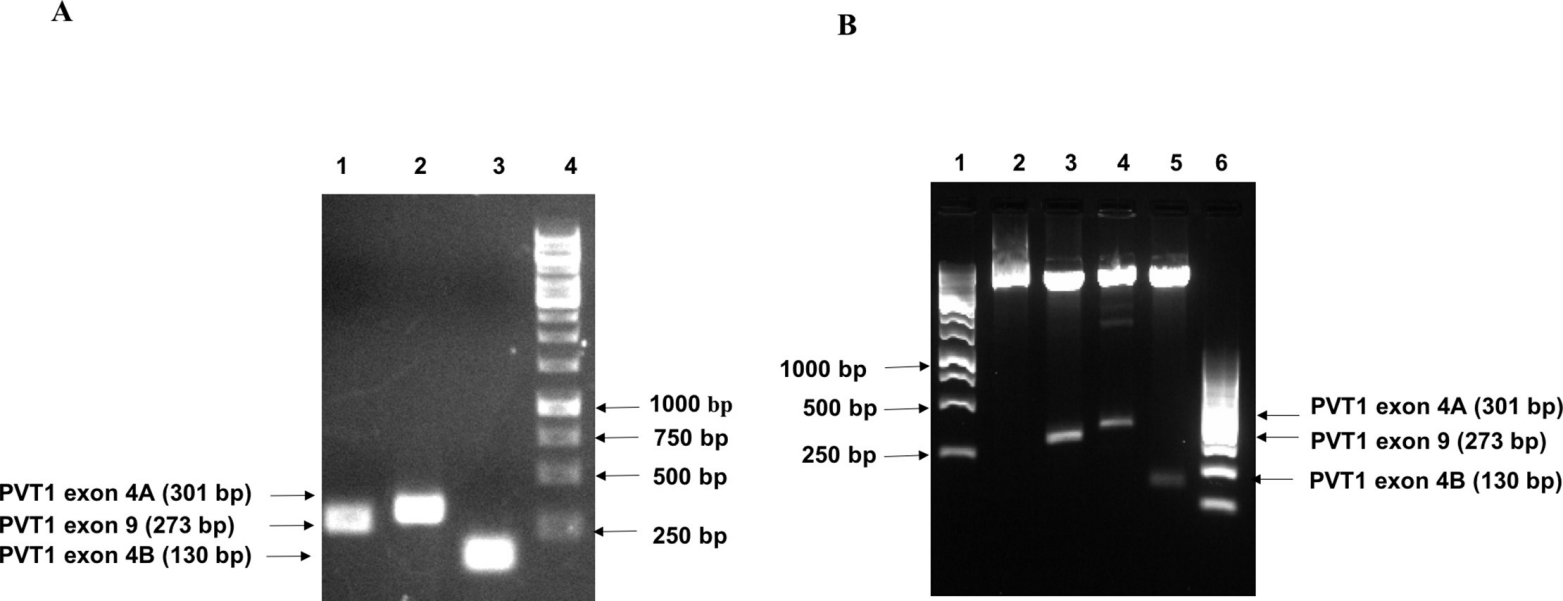
Creation of PCR standards for PVT1 exons 4A, 4B, and 9. A. Polymerase chain reaction showing PVT1 exons 4A, 4B, and 9 products [lane 1 –PVT1 exon 9, lane 2 –PVT1 exon 4A, lane 3 –PVT1 exon 4B, lane 4 –ladder (1 kb)]. B. Confirmation of PVT1 exons 4A, 4B, and 9 plasmid clones through restriction digestion [lane 1–1 kb ladder; lane 2 –vector backbone without insert; lanes 3, 4, 5 –digested plasmid showing vector backbone and PVT1 exon 9 (273bp), exon 4A (301bp), exon 4B (130bp), respectively; lane 6–100 bp ladder.

**Fig 3:**
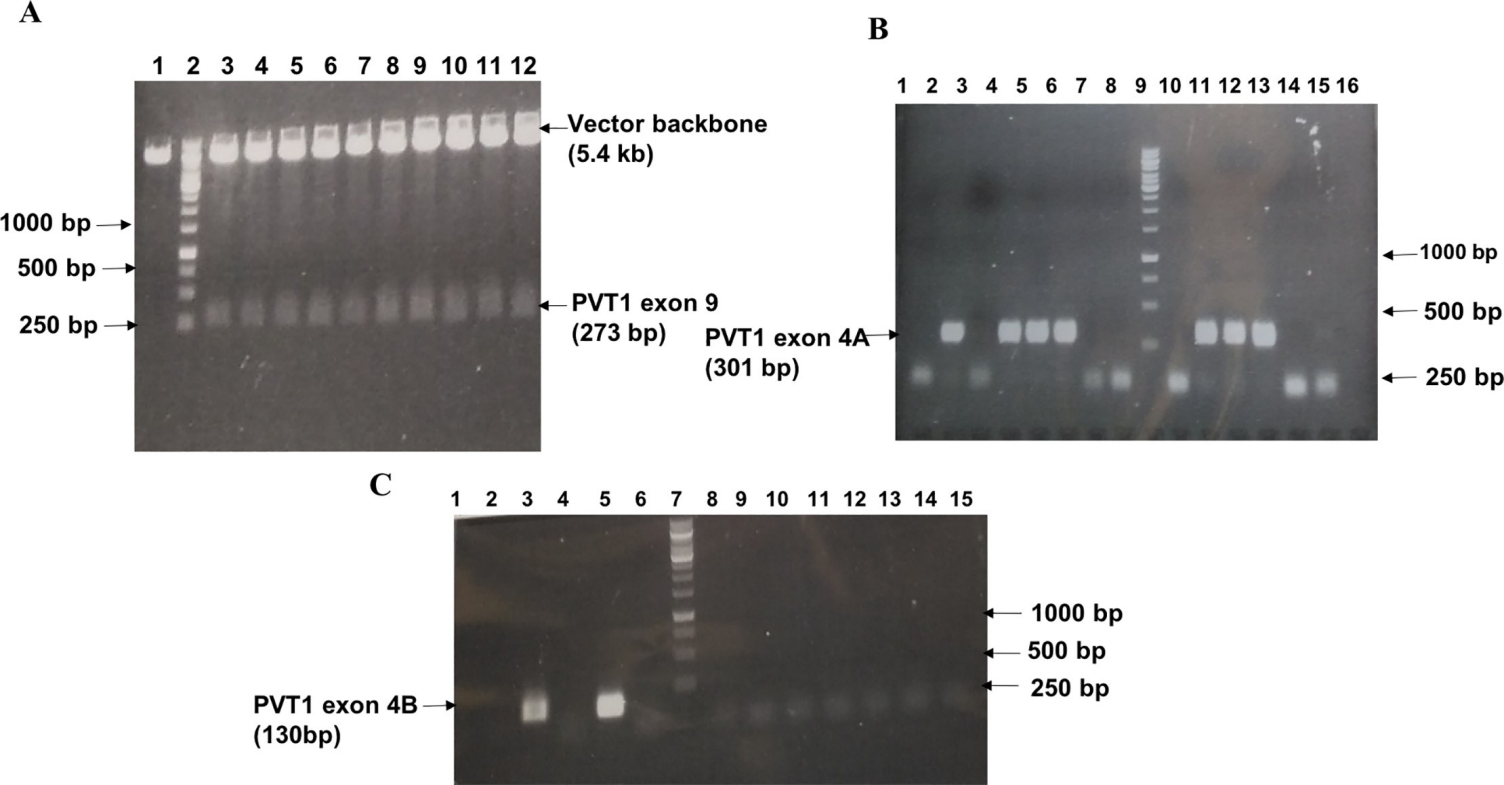
Confirmation of PCR standards for PVT1 exons 4A, 4B, and 9. A. Confirmation of PVT1 exon 9 plasmid clones through restriction digestion [lane 1- plasmid vector digested with BamHI and HindIII, lane 2- DNA Ladder (1 kb), lanes (3–12)- positive clones digested with BamHI and HindIII showing vector backbone and PVT1 exon 9 insert (273bp)]. B. Colony PCR showing the presence of PVT1 exon 4A insert (301 bp) in plasmid vector. Positive clones are present in lanes 2, 4–6, 11–13. No insert is present in lanes 1, 3, 7, 8, 10, 14, 15; lane 9–1 kb ladder; lane 16 –blank. C. Colony PCR showing the presence of PVT1 exon 4B insert (130 bp) in plasmid vector. Positive clones are present in lanes 3 and 5. No insert is present in lanes 1, 2, 4, 6, 8–14; lane 7–1 kb ladder; lane 15 –blank.

Amplification was performed on a 2720 Thermal cycler (Applied Biosystems) with an initial denaturation temperature of 94°C for 5 min, followed by 35 cycles of 94°C for 1 min, 56°C for 45 sec, 72°C for 1 min and a final extension of 10 min at 72°C. The purified product was cloned into a plasmid vector and positive clones were confirmed through colony PCR, restriction digestion (Figs 2 and 3), and finally by sequencing.

### Cell culture and cell culture reagents

The cell lines used for this study are RWPE-1 (non-tumorigenic prostate epithelial cell line, Caucasian male (CM)), WPE1-NA22 (derived from RWPE-1, indolent, androgen-dependent, CM), MDA PCa 2b (aggressive, androgen dependent, from a male of African ancestry (moAA), PC-3 (aggressive, androgen-independent, CM), LNCaP (aggressive, androgen-dependent, CM), C4-2B (derived from LNCaP, aggressive, androgen-independent, CM, and 22Rv1 (aggressive, castration resistant).

RWPE-1 cells were cultured in keratinocyte- serum free medium (SFM) supplemented with 0.05 mg/ml bovine pituitary extract (BPE), 5 ng/ml epidermal growth factor (EGF) and 1% penicillin/streptomycin. WPE1-NA22 cells were cultured in keratinocyte-SFM medium supplemented with 0.05 mg/ml BPE, 5 ng/ml EGF and 1% penicillin/streptomycin. MDA PCa 2b cells were cultured in F-12K medium supplemented with 20% fetal bovine serum (FBS), 25 ng/ml cholera toxin, 10 ng/ml mouse epidermal growth factor, 0.005 mM phosphoethanolamine, 100 pg/ml hydrocortisone, 45 nM selenious acid, 0.005 mg/ml bovine insulin. PC-3 cells were cultured in F-12K medium supplemented with 10% fetal bovine serum and 1% penicillin/streptomycin. LNCaP cells and 22RV1 cells were cultured in RPMI 1640 supplemented with 10% heat inactivated FBS, 1% penicillin/streptomycin and 10nM testosterone. C4-2B cells were cultured in DMEM supplemented with 200ml Ham’s F12, 10% heat-inactivated FBS, 1% penicillin/streptomycin, insulin (5μg/ml), triiodothyronine (13.65 pg/ml), human apo-transferrin (4.4 μg/ml), d-Biotin (0.244 μg/ml), and adenin (12.5 μg/ml).

### RNA extraction

At 75% confluency, total RNA was extracted from cell lines in 60 X 15 mm tissue culture dishes, using RNeasy Mini Kit (Qiagen, Germany, cat# 74104). After quantification with Nanodrop1000 spectrophotometer (NanoDrop, Madison, WI, USA), 500 ng of RNA was reverse-transcribed into cDNA using QuantiTect Reverse Transcription kit using random hexamers (Qiagen, Germany, cat# 205311). For the tissue, serum, and plasma samples 100ng of RNA was reverse-transcribed. We used miRCURY^TM^exosome isolation kit-serum and plasma (miRCURY) (Exiqon, Woburn, MA) for exosome isolation from human serum, mouse plasma, and human urine. For urine samples, miRCURY^TM^exosome isolation kit-Cells, Urine and CSF (miRCURY) (Exiqon, Woburn, MA) was used for exosome isolation. In the next step, RNA was isolated using miRCURY^TM^ RNA isolation kit for biofluids according to the manufacturer’s instructions.

### Quantitative reverse transcriptase polymerase chain reaction

Using the University of California, Santa Cruz (UCSC) genome browser, we carefully annotated the PVT1 gene sequence and—exons were retrieved from the analysis. The Primer3 Plus software was used to custom-design primers for PVT1 exon 4A, exon 4B, and PVT1 exon 9. The qPCR assay was performed on an ABI 7500 platform (Applied Biosystems instruments, Grand Island, NY, USA)) with 25 μl reaction volumes containing 12.5 μl SYBR Green PCR master mix (Life Technologies, Grand Island, NY, USA cat# 4309155), 0.4 μM final concentration for primers, 2.5 μl cDNA template, and 7.5 μl of water. The thermal cycle protocol used was as follows: 50°C for 2 min, 10 min initial denaturation at 95°C, and 40 cycles of 15s denaturation at 94°C, 1 min annealing at 65°C. A dissociation curve was also added at the end of the cycle.

### Construction of standard curve

Concentration of assay standards was measured spectrophotometrically using NanoDrop® ND1000 (Thermo Scientific NanoDrop Products, Wilmington, Delaware), and the measurements were recorded in units of nanograms per microliter, which was converted into copies per microliter using the following equation: ([x ng/μl × 10–9] / [pcDNA vector and 273/301/130 DNA bps × 660]) × 6.022e23 = y copies/μl. A range of serial dilutions starting from 1.5 X 10^1^–1.5 10^10^ copies/μl for assay standards were made, and using linear regression a standard curve was constructed. Copy number-based quantification was used to determine the exact number of PVT1 exons 4A, 4B, or 9 molecules. Representative melt curves are included as S1 Fig.

## Results

### Creation of standard curve

The sizes of the amplified products of PVT1 exons 4A, 4B, and 9 by conventional PCR are presented in Fig 2A. Products were of the expected size, indicating amplification of the target template. The PCR products were cloned into a plasmid vector (Fig 2B). The positive clones were confirmed by restriction digestion, colony PCR, and sequencing (Fig 3A–3C).

**Fig 4:**
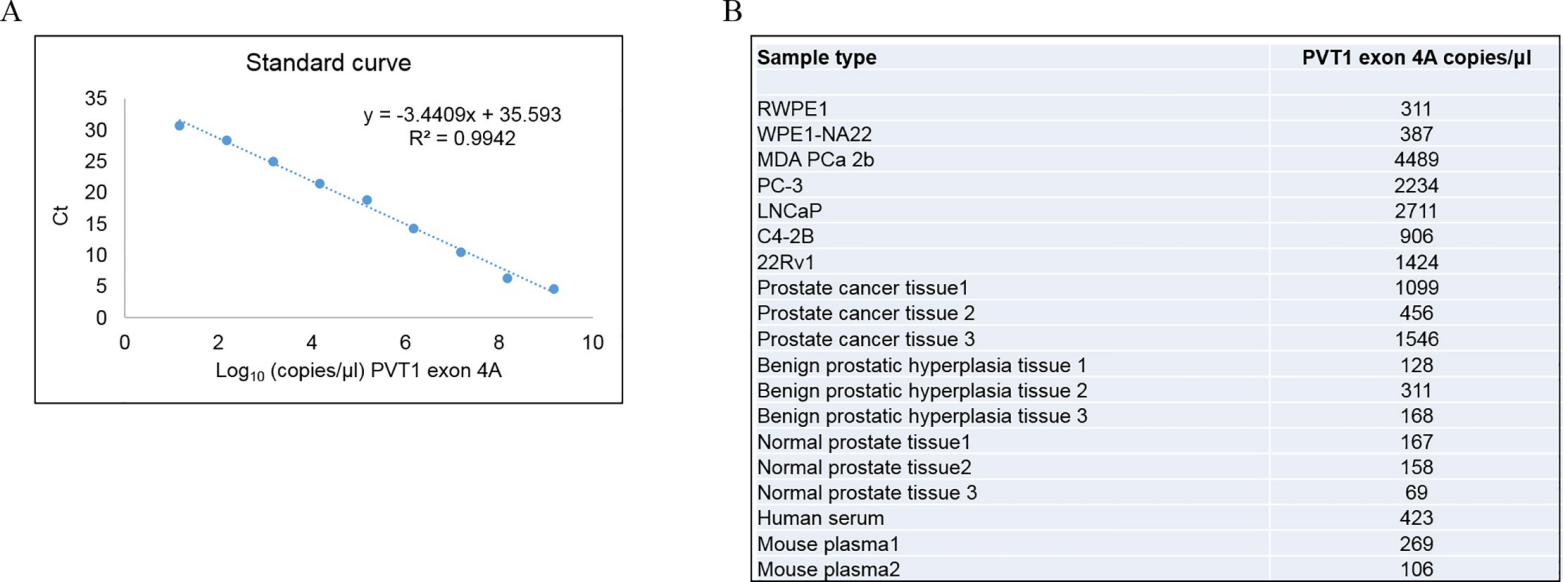
Quantitation of PVT1 exon 4A in human prostate cell lines, prostate tissues, serum, and mouse plasma. A. Standard curve for PVT1 exon 4A. B. Calculation of number of copies of PVT1 exon 4A in a variety of biological samples.

**Fig 5:**
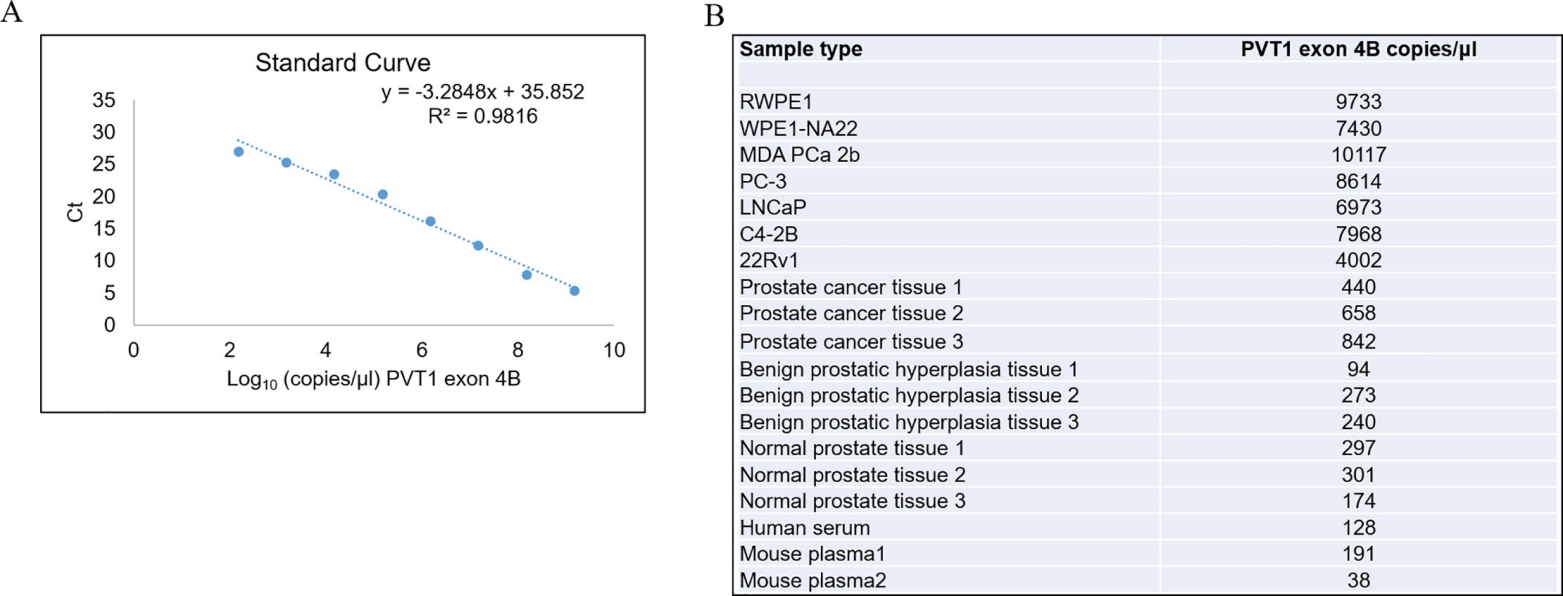
Absolute quantitation of PVT1 exon 4B in human prostate cell lines, prostate tissues, serum, and mouse plasma. A. Standard curve for PVT1 exon 4B. B. Calculation of number of copies of PVT1 exon 4B in a variety of biological samples.

**Fig 6:**
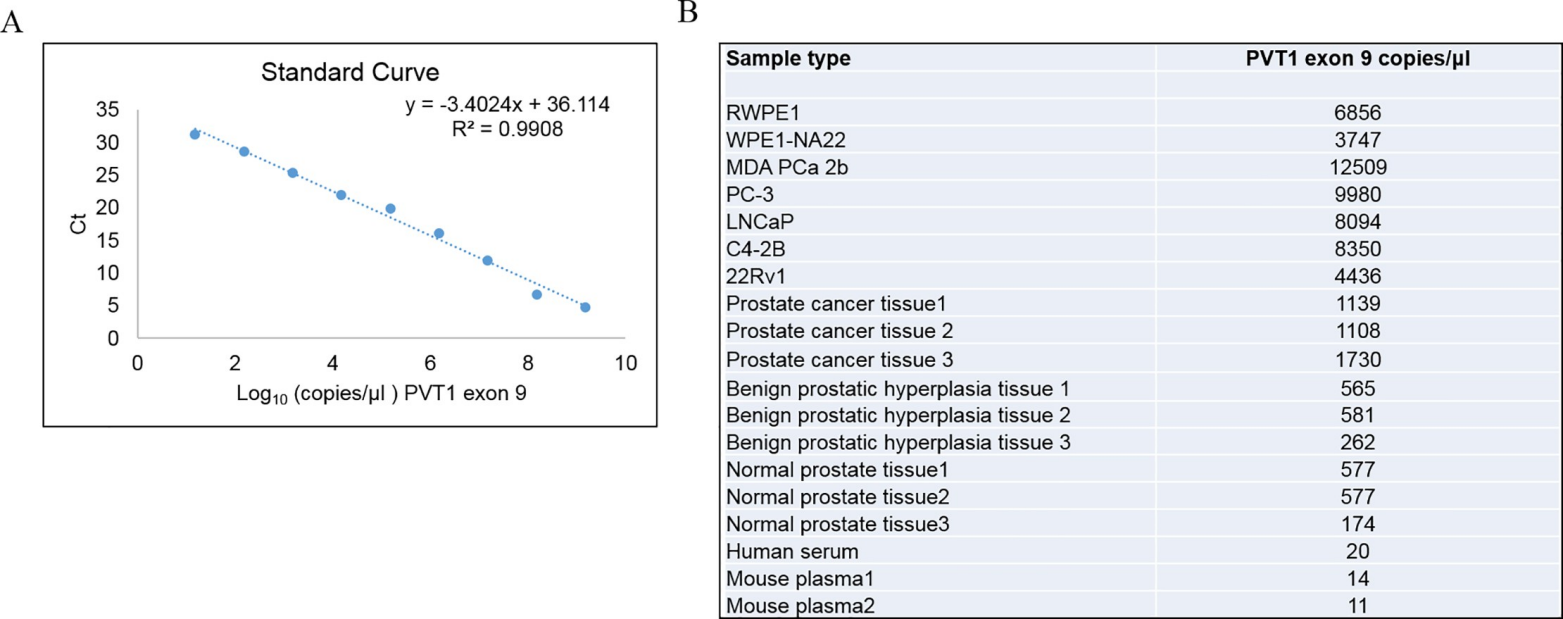
Quantitation of PVT1 exon 9 in human prostate cell lines, prostate tissues, serum, and mouse plasma. A. Standard curve for PVT1 exon 9. B. Calculation of number of copies of PVT1 exon 9 in a variety of biological samples.

For our in-house plasmids containing PVT1 exons 4A, and 4B, and 9, serial dilutions were prepared with a range 10^1^–10^10^ copies/μl-. A linear relationship was obtained between Cq values and the log10 concentrations of PVT1 exons exon 4A (301bp), exon 4B (130bp), and PVT1 exon 9 (273bp). The standard curves were used to quantify the number of copies/μl of PVT1 exons 4A, and 4B, and 9 in different prostate cancer cell lines, human prostate tissue, human serum, mouse plasma, and human urine. The standard curves of PVT1 exons 4A, and 4B, and 9 were constructed (Figs 4A–6A).

The regression analysis yielded a correlation co-efficient of 0.9942, 0.9816, and 0.9908, and the values of Y-intercept are 35.593, 35.852, and 36.114 for PVT1 exons 4A, 4B, and 9 respectively. The slopes of -3.441, -3.285, and -3.402, closely approximate the theoretical maximum efficiency.

### Detection and quantification of PVT1 exons 4A, 4B, and 9 in prostate epithelial and prostate cancer cell lines

For this study nine prostate epithelial cell lines (RWPE1, WPE1-NA22, MDA PCa 2b, PC3, LNCAP, C4-2B, 22Rv1) were used, to capture the diversity of clinical characteristics notable in PCa. The copies/μl values were calculated with the help of this standard curve and they are listed in (Figs 4B–6B). The number of copies of PVT1 transcripts were higher in MDA PCa 2b in comparison to the other cell lines.

### Detection and quantification of PVT1 exons 4A, 4B, and 9 in normal and cancerous human prostate tissue

We used the assay to assess the expression of PVT1 exons 4A, 4B, and 9 in tissue samples. PVT1 exons 4A, 4B, and 9. The expression of these transcripts were higher in prostate cancer tissues, in comparison to normal prostate and benign prostatic hyperplasia tissues (Figs 4B–6B).

### Detection and quantification of PVT1 exons 4A, 4B, and 9 in normal and cancerous human serum and mouse plasma

PVT1 exons 4A, 4B, and 9 were detectable and quantifiable in human serum and mouse plasma samples. As for detection and quantification of PVT1 exons 4A, 4B, and 9 in cell lines and tissues, the standard curves were calculated for these transcripts, and number of copies/μl of exons 4A, 4B, and 9 in human serum and mouse plasma was calculated (Figs 4B–6B).

### Detection and quantification of PVT1 exons 4A, 4B, and 9 in human urine

PVT1 exons 4A, 4B, and 9 were detectable and quantifiable in human urine samples. The standard curves were constructed for the three transcripts and then the copies/μl were calculated (Table 2). The copy number of these transcripts was higher in the samples of individuals with a history of cancer as compared to urine samples of healthy individuals.

**Table 2:** Quantification of PVT1-derived transcripts in urine samples.

Sample	Gender	Race	Current cancer	PVT1 exon 4A copies/μl	PVT1 exon 4B copies/μl	PVT1 exon 9 copeis/μl
Sample 1	Female	Black	No	173	83	73
Sample 2	Female	Latino	No	140	1182	83
Sample 3	Female	Black	No	55	131	49
Sample 4	Male	Black	No	1415	79	220
Sample 5	Female	Black	No	1854	772	54
Sample 6	Male	Latino	No	387	133	151
Sample 7	Female	Latino	No	736	71	670
Sample 8	Female	Latino	No	151	98	418
Sample 9	Male	White	Yes	1804	1933	2855
Sample 10	Male	Black	Yes	1144	2613	2619

### Assessment of precision and accuracy using different cell lines

To assess precision and accuracy of the assay, we compared PVT1 exons 4A, 4B, and 9 in cell lines with previously known expression profiles of PVT1 exons 4A, 4B, and 9: (1) RWPE1 (with low expression of PVT1), RWPE1_ex4A (with overexpression of PVT1 exon 4A), (2) RWPE1_ex4B (with overexpression of PVT1 exon 4B), and (3) RWPE1_ex9 (with overexpression of PVT1 exon 9). As expected, using same volumes of cDNA of the same concentrations we observed that RWPE1_ex4A had higher copy numbers of PVT1 exon 4A, and RWPE1_ex4B had higher copy numbers of PVT1 exon 4B, and RWPE1_ex9 had higher copy numbers of PVT1 exon 9 than RWPE1 (Table 3). We also made three serial dilutions of the cDNA samples. And we found that for RWPE1, RWPE1_ex4A, RWPE1_ex4B, and RWPE1_ex9 the transcript copy numbers detected in serial dilution 1 was always more than that detected in serial dilution 2 and the copy numbers detected in serial dilution 2 was always more than the transcript copy numbers detected in serial dilution 3 (Table 3). We also used GAPDH as a housekeeping gene to independently assess the amount of template RNA input used in each experiment. The Cq values for GAPDH were very much similar for all the samples used in the same experiment, thus indicating that amount of template RNA input used was identical. Representative GAPDH data is shown in S1 Table.

**Table 3:** Quantification of PVT1 exons 4A, 4B, and 9 transcripts copy numbers in cell lines with known PVT1 exons 4A, 4B, and 9 expression profiles.

Transcript	Sample	Copies/μl	Sample	Copies/μl
PVT1 exon 4A	RWPE1_ex4A_dilution 1	4790	RWPE1_dilution 1	126
	RWPE1_ex4A_dilution 2	1824	RWPE1_dilution 2	61
	RWPE1_ex4A_dilution 3	754	RWPE1_dilution 3	32
PVT1 exon 4B	RWPE1_ex4B_dilution 1	12299	RWPE1_dilution 1	4135
	RWPE1_ex4B_dilution 2	5096	RWPE1_dilution 2	1694
	RWPE1_ex4B_dilution 3	2702	RWPE1_dilution 3	643
				
PVT1 exon 9	RWPE1_ex9_dilution 1	6096	RWPE1_dilution 1	2662
	RWPE1_ex9_dilution 2	2423	RWPE1_dilution 2	1029
	RWPE1_ex9_dilution 3	958	RWPE1_dilution 3	392

## Discussion

Long non-coding RNAs (lncRNAs) have attracted much attention due to their large number and biological significance [34]. LncRNAs are important targets for cancer diagnostics and therapeutics due to their critical role in numerous cellular processes. PVT1, a long non-coding RNA, has been designated as an oncogene due to its contribution to the phenotype of multiple cancers. PVT1 overexpression has been demonstrated in pancreatic cancer, lung cancer, gastric cancer, and is related to poor prognosis in most of these cases [35–37]. PVT1 is also associated with immune diseases like asthma, vitiligo, and several others [17, 38]. And PVT1 has been demonstrated by multiple studies and research groups to be overexpressed in prostate cancer [32,39]. PVT1 plays an oncogenic role and regulates tumor growth in prostate cancer [18]. PVT1 promotes prostate cancer invasion and metastasis by modulating epithelial to mesenchymal transition [39]. Evidence has accumulated that PVT1 could be used as potential biomarker for prostate cancer [40].

The first prostate cancer biomarker was prostatic acid phosphatase (PAP), which was a clinical marker for prostate cancer progression. PAP was later replaced by PSA and PCA3. Owing to different inherent limitations all of these, the field of prostate cancer biomarkers is evolving rapidly. A biomarker may be used as a tool for disease diagnosis and prognosis, and for predicting and monitoring clinical response to an intervention [15,40]. In this study, we have created a qPCR-based assay to reliably and reproducibly detect and quantify transcripts derived from PVT1 exons 4A, 4B, and 9 in a variety of biological samples, including urine, serum and plasma.

Quantitative PCR (qPCR)-based assays have routinely been used for the detection of hepatitis B virus and hepatitis C virus [41]. There is no report of any quantification assay for detection of any PVT1-derived transcripts. In this study, we developed a qPCR-based assay for the quantification of PVT1 exons 4A, 4B, and 9 in a variety of mouse and human biological samples, including cell lines, tissues, serum, mouse plasma, and human urine. The primers used for detecting PVT1 exons 4A, 4B, and 9 are specific for the detection of these highly conserved regions of the PVT1 gene.

To demonstrate the effectiveness of the qPCR-based assay developed, samples from non-tumorigenic prostate epithelial cell line, prostate cancer cell lines, normal prostate tissues, benign prostatic hyperplasia tissues, prostate cancer tissue, human serum, mouse plasma, and human urine samples were tested. The qPCR showed a close correlation between Cq and log10 values of PVT1 exons 4A, 4B, and 9 ranging from 10^1^ to 10^10^ copies/reaction. The testing of cell lines showed that PVT1 exons 4A, 4B, and 9 are significantly and consistently overexpressed in aggressively tumorigenic cell line MDA PCa 2b that we had previously reported to have very clear overexpression of PVT1 [30]. Further, testing of human prostate tissue samples showed that the number of copies/μl of PVT1 exons 4A, 4B, and 9 is higher in prostate cancer samples in comparison to normal prostate tissues or benign prostatic hyperplasia tissues. These absolute quantitative data agree with the relative expression data we had previously acquired [31], [32]. It is noteworthy that using this novel copy number–based quantification assay, we were able to detect and quantify PVT1 exons 4A, 4B, and 9 in both serum and plasma samples. These data clearly demonstrate the ability of this assay to detect these transcripts in a wide variety of biological samples. In addition to serum and plasma, this assay can also detect and quantify PVT1 exons 4A, 4B, and 9 in urine. The copy number of PVT1 exons 4A, 4B, and 9 is higher in urine samples of individuals with history of cancer in comparison to urine samples of individuals without history of cancer. The current study has established copy number–based quantification of PVT1 exons 4A, 4B, and 9 transcripts in biological samples, especially urine. Previous studies have utilized relative quantification of PVT1 mRNA expression, but they have not specified which alternatively spliced PVT1 transcripts they quantified [42]. To our knowledge, this is the first report of a copy number–based assay that quantifies alternatively spliced transcripts from PVT1 exons 4A, 4B, and 9.

In a future study, we will use statistically-powered sample size to determine the sensitivity, specificity, precision, and accuracy of the assay. And we will include adequate numbers of normal prostate tissue samples and prostate cancer tissue samples, as well as urine and blood samples from both normal healthy individuals and prostate cancer patients.

Consequently, based on its ability to detect and quantify PVT1 exons 4A, 4B, and 9 in serum and plasma, and in urine, this is an assay with potential non-invasive applications in diseases where PVT1 is dysregulated. Moreover, based on the fact that this assay predictably found the cell lines overexpressing PVT1 exon 4A (RWPE1_ex4A), PVT1 exon 4B (RWPE1_ex4B), and PVT1 exon 9 (RWPE1_ex9) to have more copy numbers of PVT1 exons 4A, 4B, and 9, respectively, than RWPE1 and that dilution resulted in detection of proportionally less respective PVT1 exons, this assay demonstrates evidence of accuracy and precision.

One important aspect of this assay is that it can detect and quantify both low and high copy numbers of PVT1 exons 4A, 4B, and 9. Joshi *et al* and Ballester *et al* have previously described assays that were used to determine the copy numbers of transcripts in transgenic animals and they had set their thresholds for low copy numbers as 10 [43–44]. In the current study, we have used a variety of different types of biological samples, such as, cell lines, tissues, plasma, serum and urine. The amount of RNA used for making cDNA from cell line samples was 500ng and the amount used for other “difficult to obtain” samples such as tissues, plasma and urine was 100ng strictly because of their limited availability. Based on our data from the abundant samples (cell lines), we have set 10^3^ copies as the threshold for determining low or high copy numbers for when 500ng of template RNA is used to make cDNA for the performance of this novel assay. Another noteworthy aspect of this assay is its amplification efficiency, which is stable over a range of input copy numbers.

These characteristics will likely lend it to robust clinical usage. With the development of this copy number-based quantification assay for non-invasive detection of PVT1-derived transcripts, the stage is now set for testing specific usage in a variety of clinical settings via the performance of multi-center clinical trials. In addition, we will set up this assay in a CLIA-certified clinical laboratory, and create standard operating procedures, appropriate quality control ranges. Further, we will require multiple laboratory technologists to test the assay using routine clinical laboratory workflows, and they will be required to prepare formal clinical laboratory reports with appropriate reference ranges and accompanying interpretation.

In conclusion, in this paper, we report the development of a novel qPCR-based assay for detection and copy number–based quantification of PVT1 exons 4A, 4B, and 9 in a variety of biological samples including blood and urine. This assay may have non-invasive applications in prostate cancer and other diseases where PVT1 is dysregulated.

## Supporting information

S1 Fig Melt curve plot of PVT1 exons 4A, 4B, and 9. (TIFF)

S1 File Raw gel pictures. (DOCX)

S1 Table Representative Cq values demonstrate comparable RNA template input. (DOCX)
