# Supplementary figures and images for: Copy number-based quantification assay for non-invasive detection of PVT1-derived transcripts
Source: PLoS One. 2019 Dec 26;14(12):e0226620. doi: 10.1371/journal.pone.0226620 (PMC6932808; doi:10.1371/journal.pone.0226620)

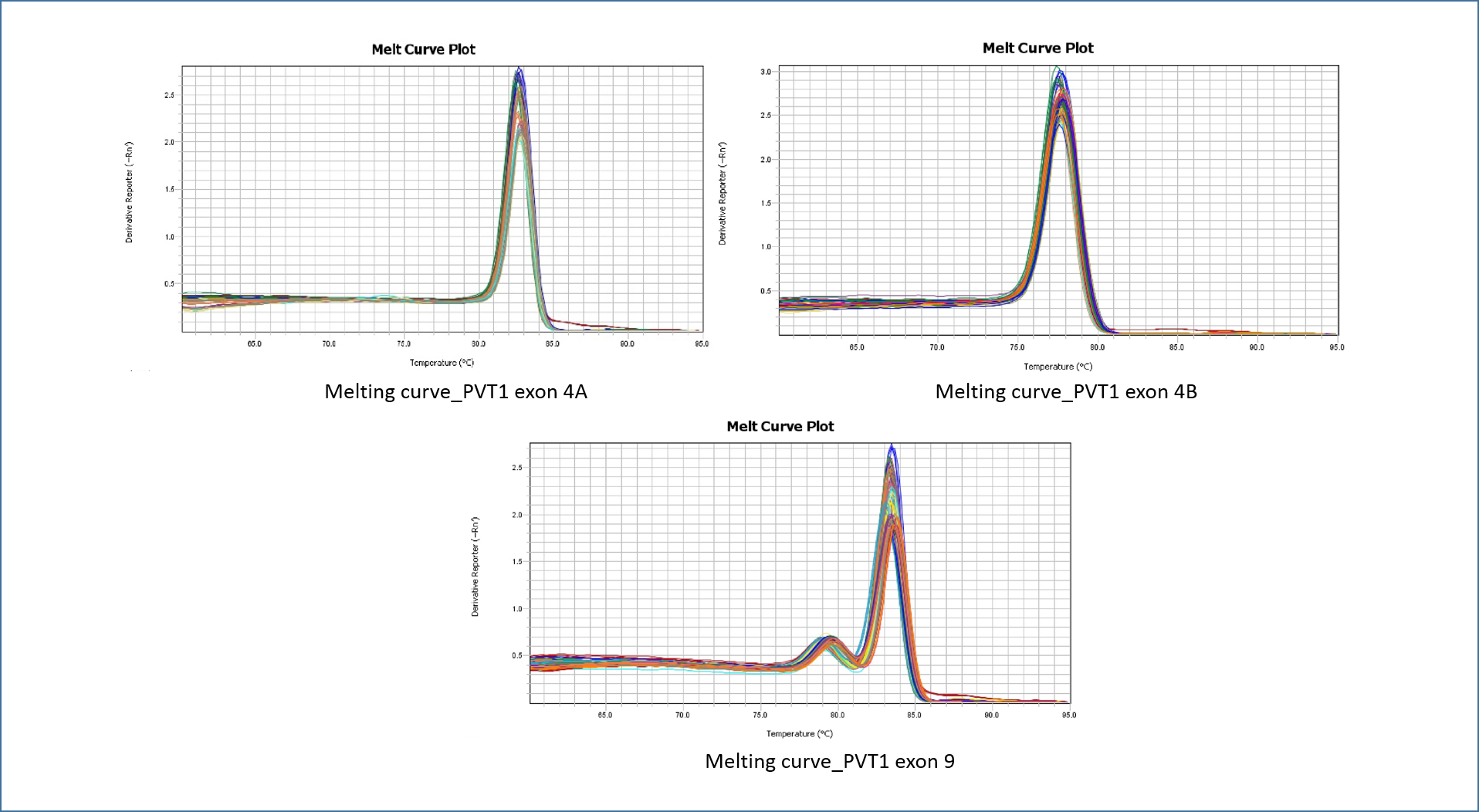

Supplement: S1 Fig — (TIFF) [file pone.0226620.s001.tiff]
