# Supplementary material for: Copy number-based quantification assay for non-invasive detection of PVT1-derived transcripts
Source: PLoS One. 2019 Dec 26;14(12):e0226620. doi: 10.1371/journal.pone.0226620 (PMC6932808; doi:10.1371/journal.pone.0226620)

**Supplementary gel electrophoresis raw data for Figure 2: Creation of PCR standards for PVT1 exons 4A, 4B, and 9. A. Polymerase chain reaction showing PVT1 exons 4A, 4B, and 9 products B. Confirmation of PVT1 exons 4A, 4B, and 9 plasmid clones through restriction digestion**


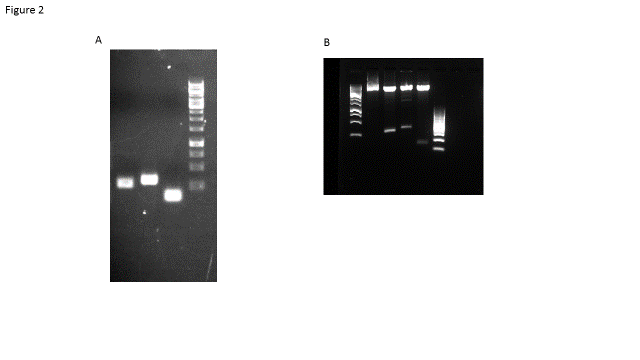


**Supplementary gel electrophoresis raw data for Figure 3: Confirmation of PCR standards for PVT1 exons 4A, 4B, and 9. A. Confirmation of PVT1 exon 9 plasmid clones through restriction digestion. B. Colony PCR showing the presence of PVT1 exon 4A insert (301 bp) in plasmid vector. C. Colony PCR showing the presence of PVT1 exon 4B insert (130 bp) in plasmid vector.**

**A B**


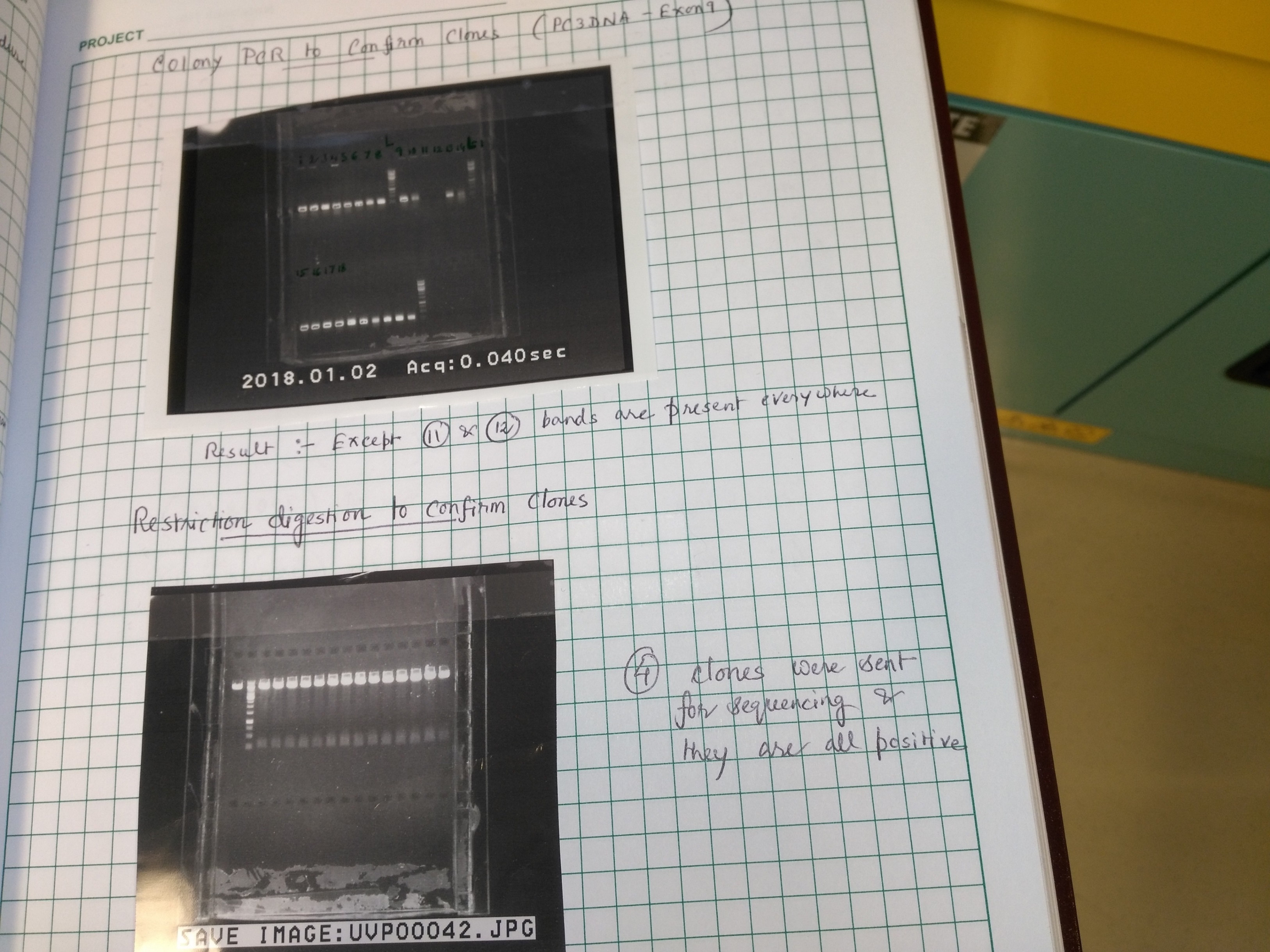

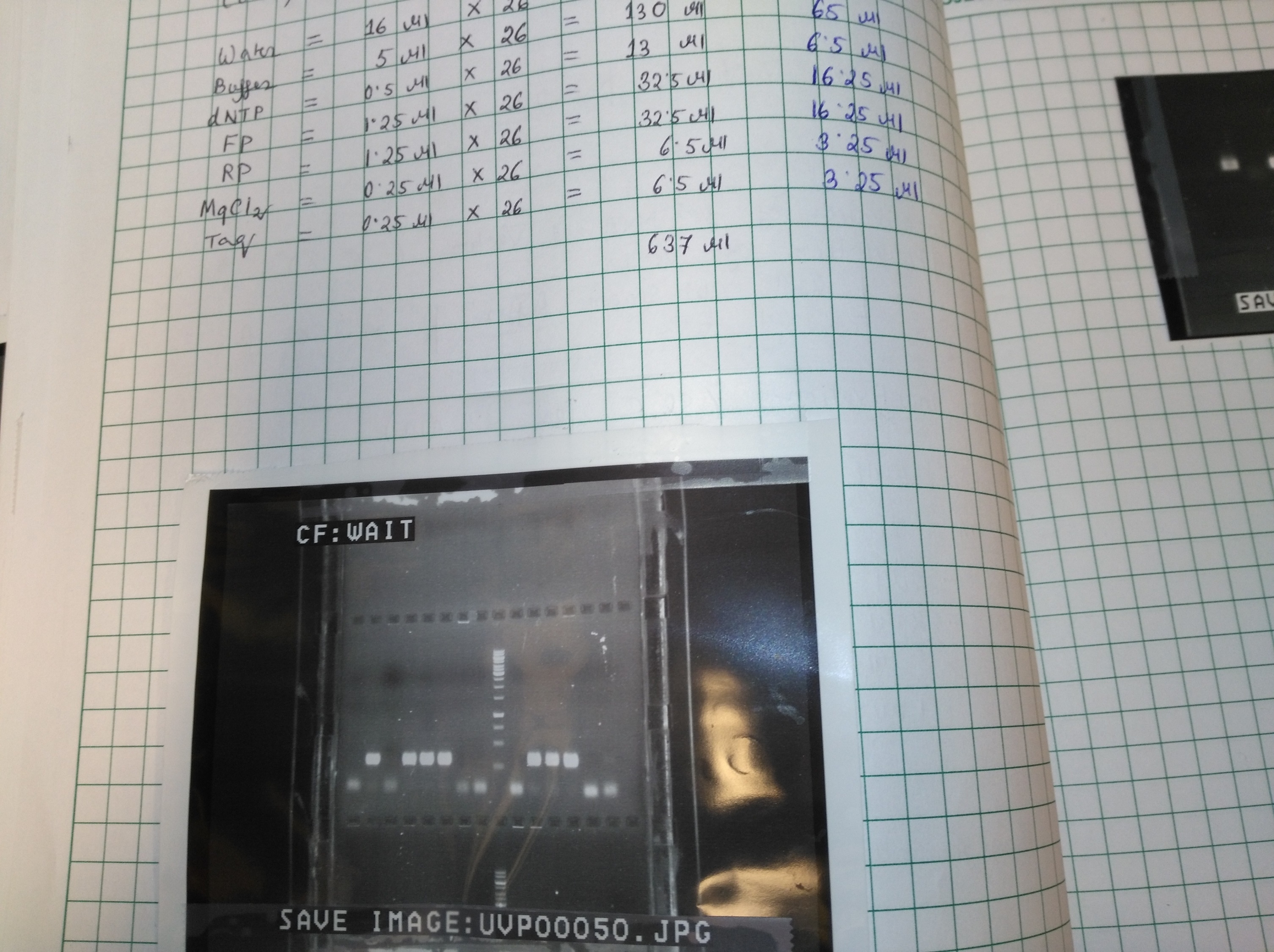


**C**


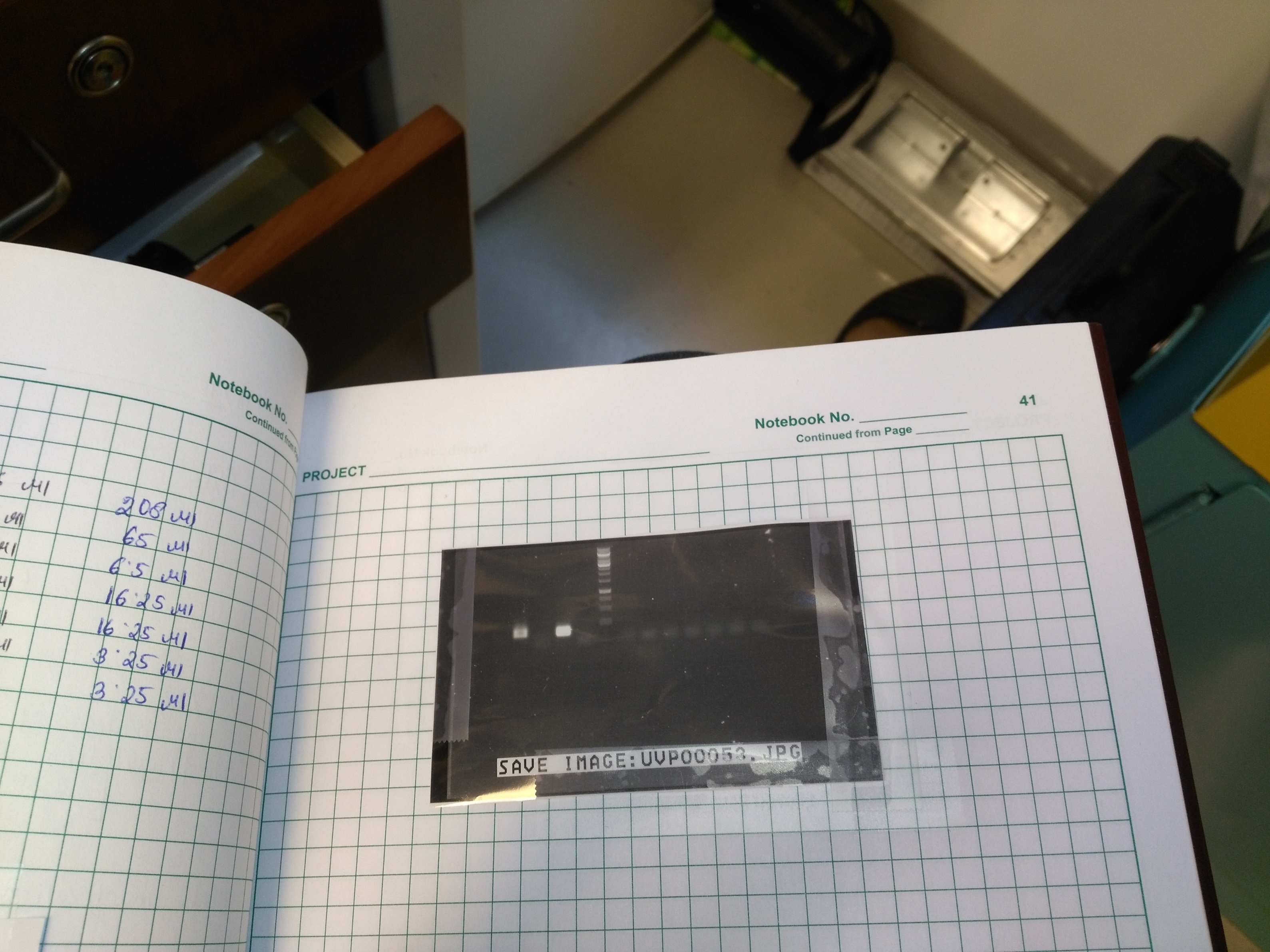

Supplement: S1 File — (DOCX) [file pone.0226620.s002.docx]
