# Supplementary material for: Copy number-based quantification assay for non-invasive detection of PVT1-derived transcripts
Source: PLoS One. 2019 Dec 26;14(12):e0226620. doi: 10.1371/journal.pone.0226620 (PMC6932808; doi:10.1371/journal.pone.0226620)
Supplement: S1 Table — (DOCX) [file pone.0226620.s003.docx]

**Supplementary Table: Representative Cq values demonstrate comparable RNA template input**

|  | PVT1 exon 9 Primer | | GAPDH Primer | |
| --- | --- | --- | --- | --- |
|  | RWPE1_ex9 | RWPE1 | RWPE1_ex9 | RWPE1 |
| Dilution 1 | 21.622 | 22.655 | 18.497 | 18.543 |
| Dilution 2 | 22.771 | 23.839 | 19.510 | 19.538 |
| Dilution 3 | 23.929 | 25.043 | 20.501 | 20.487 |
